# Emotional Intelligence and Social Support: Two Key Factors in Preventing Occupational Stress during COVID-19

**DOI:** 10.3390/ijerph18136918

**Published:** 2021-06-28

**Authors:** Giusy Danila Valenti, Palmira Faraci, Paola Magnano

**Affiliations:** Faculty of Human and Social Sciences, University of Enna Kore, 94100 Enna, Italy; palmira.faraci@unikore.it (P.F.); paola.magnano@unikore.it (P.M.)

**Keywords:** occupational stress, emotional intelligence, social support, COVID-19

## Abstract

Emotional intelligence (EI) and social support are among the most investigated hypothesized variables that affect stress at work. The current study aims to evaluate the direct association between EI and occupational stress and its indirect relationship mediated by three sources of social support during the spread of the COVID-19. The total sample was composed of 367 individuals (53.7% males), aged from 20 to 68 (*M* = 37.84, *SD* = 10.39), who filled out an online questionnaire. A mediation analysis was performed to test the hypothesized relationships. Our findings showed that EI has a direct effect on psychological effects and an indirect effect on almost all the facets of occupational stress. The significant mediators were social support from both family and friends. Theoretical and practical implications are discussed and directions for future studies are suggested.

## 1. Introduction

Occupational stress, also named work stress, is a psychological and physiological phenomenon, generated as a response to various external factors [1]. Resulting from insufficient coping skills with stressors at the workplace, occupational stress is a negatively perceived quality and has negative consequences on mental and physical health. This means that, prior to showing stress symptoms, at first, individuals must perceive a stressor negatively and then they must display inadequate coping abilities. That is, if a source of stress is perceived as a challenge to overcome rather than a threat to avoid, no negative outcomes will appear on mental and physical health [2].

Findings from previous studies have showed associations between high levels of stress at work and a broad range of disturbances, such as chronic fatigue, eating disorders, increased blood pressure, and the risk of cardiovascular diseases. Regarding psychological symptoms, occupational stress has been linked to depression and anxiety, mood disturbance and emotional exhaustion, and a decrease in attention and concentration [3,4,5,6,7].

In addition, significant positive associations were also found between occupational stress and a variety of job-related outcomes, such as intention to leave the workplace or absenteeism [6,8], whereas inverse relationships were estimated with job satisfaction, job performance, job motivation, and organizational commitment [9].

Occupational stress is considered both as a “public concern” and a “personal trouble” [10], because both job-related and individual factors influence it. Regarding job-related factors, some studies have linked occupational stress to several aspects, such as heavy workload, role ambiguity, role conflict, problematic interactions with colleagues or supervisors, inadequate training, job insecurity, low salary, and lack of career prospects [8,11,12].

With regard to individual factors, several studies have revealed significant associations with gender, age, educational level [13,14,15], and coping styles [16].

Owing to the complexity and heterogeneity of occupational stress, consensus about its assessment is lacking. Though some authors, such as Mensah [17], used a single item simply asking people “*Do you experience stress at work?*”, occupational stress is described as a multidimensional construct, and overload, work relations, psychological symptoms and physical burdens, pay and benefits, lack of rewards, and organizational policies are among the most widely investigated indicators [8,18,19].

A relevant individual factor related to occupational stress is emotional intelligence (EI), defined as a personality predisposition associated with individuals’ tendency to understand their own and others’ emotions, to manage their own feelings and their relationships with others [20]. Understanding emotions helps people to be aware of their own and others’ behaviors and motivations, whereas managing emotions allows the individuals to navigate their feelings constructively at work. In other words, EI is the individuals’ ability to properly handle their own interpersonal and intrapersonal skills, which improves the competence in facing stressors and, consequently, enhances positive outcomes. In addition, Goleman [21] asserts that EI is twice as important as technical skills and more important than IQ in predicting positive outcomes at the workplace, suggesting that people should be judged not according to their own intelligence or professional competence, but rather by their own behaviors toward themselves and others. These premises stress the relevance of taking into account EI in working environments, both to increase productivity and efficiency and to improve workers’ wellbeing, job motivation, and job satisfaction.

Indeed, recent studies [18,22,23,24,25,26,27,28] have reported that workers with higher EI are more productive at the workplace and can cope with stressors more efficiently. The inverse relationship between EI and occupational stress has been found in different working contexts and for different categories of workers, such as police officers [26], human service professionals [19], bank employees [18], managers [29], health care professionals [30], and college teachers [31]. These findings outline that EI negatively affects occupational stress, regardless of the specific working sector. Although there is wide agreement about the negative association between EI and stress at the workplace, some authors did not show any statistical relationships [32], suggesting that other variables, such as organizational support, are protective factors in stress management rather than EI.

Social support has long been identified as a crucial resource for mitigating threats and challenges [33,34]. It is defined as the extent to which people perceive others as attentive and responsive to their needs. Social support is considered as an important factor in maintaining wellbeing and coping with challenges [35]. It can be assessed as both a global and generalized perceived social support and by discriminating different sources, such as social support from family, friends, and significant others [36,37,38,39]. However, in the work context, work-related social support (social support from coworkers and/or supervisors) is mostly investigated because these individuals are considered as the main sources of social support for workers seeking to accomplish their goals and adjust to the workplace [40]. Actually, the results of the studies investigating the effects of work-related social support on the levels of occupational stress are incoherent and inconsistent, suggesting that the kind and the quality of interactions with coworkers and supervisors may function both as protective and risk factors [11,41]. Nevertheless, a limited number of studies examining the relationships between sources of social support and occupational stress outlined the beneficial role in mitigating the degree of stress at the workplace [42,43].

Individuals who are able to understand their own and others’ feelings more likely search for support from others in challenging situations [44]. Specifically, they may need others to empathize with their situation, identify their emotional reactions, and provide social support or resources to deal with a stressful situation [45]. Social support is a key candidate to mediate EI and wellbeing. Some theorists suggested that emotional abilities contribute to acquiring social skills, thus enhancing both the quality of relationships and the availability of social support, which in turn leads to a richer sense of wellbeing [46]. Some evidence supported this hypothesis. For example, some authors showed that people with high EI reported greater social support, as well as higher levels of satisfaction and lower grades of psychological distress [36,37,38,39,47,48]. Nevertheless, the mediating role of social support in the relationship between EI and occupational stress has not yet been explored. This study aims to fill this gap.

The diffusion of the COVID-19 virus has considerably affected work conditions, leading to new job demands and pressures. Though some working sectors—such as health care professionals—are more vulnerable to occupational stress, given the higher risk of being infected and longer working hours, the current pandemic has greatly influenced each working sector without distinction. In fact, many workers have experienced—and are experiencing—different changes at work, involving an increase or a reduction of working hours, alterations in job tasks and shifts, and a transition toward smart working. In other words, many working sectors have reorganized their environments and structures to accommodate the emerging demands. All these factors may further influence how people feel in their workplaces and affect their level of occupational stress.

A large number of studies are currently examining how the pandemic is changing work conditions and affecting several job-related outcomes [17,49,50,51]. Among them, some authors have pointed out that both EI and social support have a strong impact in mitigating negative job-related outcomes. For example, Soto-Rubio et al. [52] have emphasized EI’s key role in preventing burnout among health care professionals and in improving their levels of job satisfaction, whereas other authors [53] have stressed the influence social support has in enhancing job engagement and job retention intention.

**Figure 1:**
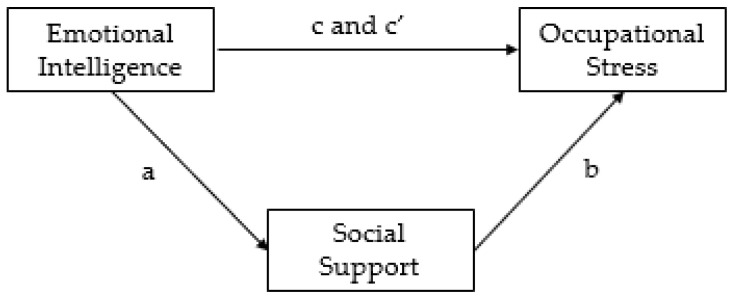
Conceptual model. Path a = association between EI and social support; path b = association between social support and occupational stress; path c = total effect of EI on occupational stress; c′ = direct effect of EI on occupational stress.

In summary, the relationship between EI and stress has been widely studied, as well as the beneficial role of social support in maintaining health and wellbeing. Further, their protective role in decreasing the levels of stress at work is well documented. However, the joint contribution of EI and social support in reducing occupational stress has not been examined during the COVID-19 lockdown. Given the importance of EI and social support in preventing occupational stress, this study aims to analyze these relationships in the Italian context during the pandemic. Specifically, the goals of this work are (a) to examine the direct relationship between EI and occupational stress, and (b) to test the mediating role of social support (see Figure 1 for a visual representation of our hypotheses). We expect that individuals with higher levels of EI will perceive their work environment as less stressful and will experience less negative health consequences, and that social support can function as a buffer in the relationship between EI and occupational stress. Thus, we formulated the following hypotheses: (i) EI negatively affects occupational stress and (ii) social support mediates the association between EI and occupational stress. Although a similar mediation analysis has not been previously tested, the proposed model derives from the existing literature described above in which the associations between EI and social support, between social support and occupational stress, as well as between IE and occupational stress have been investigated [18,19,28,29,30,31,42,43,44,46].

**Figure 2:**
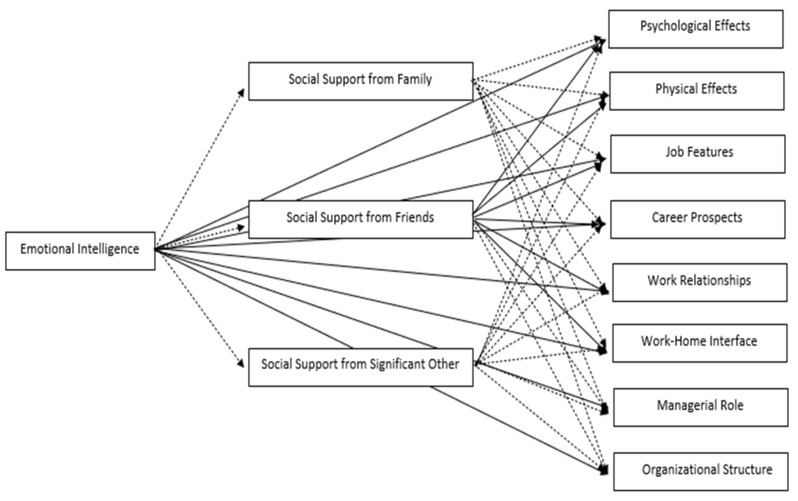
Hypothesized relationships among the investigated variables. Full lines indicate direct effects; dotted lines indicate indirect effects.

The current study takes into account support from family, friends, and significant others—which are little investigated in this specific field of study—to explore how not work-related sources of support affect occupational stress. This latter is defined as a broad concept in which effects on health (both psychological and physical) and work stressors (job features, career prospects, managerial role, work relationships, work–home interface, and organizational structure) are indicators. Figure 2 displays the hypothesized relationships among the investigated variables.

This research contributes to a better understanding of job-related outcomes in the current circumstances.

## 2. Materials and Methods

### 2.1. Participants

A sample of 385 individuals was recruited to fill out an online questionnaire. The inclusion criteria were age >18 and being employed. The initial screening led to eliminating 18 participants owing to their failure to complete the whole survey. We retained participants for subsequent analyses if they reported a small number of missing data, which were handled by replacing them with the mean score imputation for each considered variable. The final sample was composed of 367 individuals, aged from 20 to 68 (*M* = 37.84, *SD* = 10.39), almost equally distributed between the two genders, mostly married, and with higher education. Almost half of them (47.1%) declared not to have children, whereas the remaining (52.9%) reported having from one to five children. Participants were asked to indicate how/where they had been working during the last year (work remotely only/work remotely, but also at the workplace/work at the workplace, but also remotely/work at the workplace only), and to report the extent to which their working conditions changed after the spread of the pandemic (from “Not at all” to “Very much”). They were also asked to specify which (if any) working conditions changed, choosing the suited answers among multiple alternatives (salary increase/salary decrease, working hours increase/working hours decrease, kind of job activity, layoff, relationships with coworkers and supervisors) (see Table 1 for a more detailed description of the study sample).

### 2.2. Procedure

Data were gathered online, sharing the research link on social media, such as Facebook and LinkedIn, and through personal contacts. The introduction to the questionnaire included the researchers’ institutional identity, a short explanation about the aim of the study, and an invitation to participate. Individuals were informed that their participation in the study was voluntary, and they were also assured of the confidentiality of the information obtained. Informed consent was obtained by all participants prior to answering the survey. Data were collected in February 2021. All procedures were performed in compliance with provisions from the Declaration of Helsinki regarding research on human participants, approved by the Internal Review Board of Research in Psychology of UKE (UKE-IRBPSY-03.21.02).

### 2.3. Measures

#### 2.3.1. Demographics

Demographics were assessed using an ad hoc measure.

#### 2.3.2. Emotional Intelligence

The Self-Report Emotional Intelligence Test (SREIT; [54,55]) was used to assess emotional intelligence. It is a 33-item scale (e.g., “*Emotions are one of the things that make my life worth living*”; “*I am aware of my emotions as I experience them*”) on a five-point Likert scale from 1 “strongly disagree” to 5 “strongly agree”. It is a unidimensional scale, with higher scores indicating a greater level of EI. Cronbach’s alpha reached 0.93.

#### 2.3.3. Social Support

To measure perceived social support, the Italian version [56] of the Multidimensional Scale of Perceived Social Support (MSPSS; [57]) was used. The scale is composed of 12 items (e.g., “*I can tell about my problems with my family*”*;* “*My friends really try to help me*”) with response options on a six-point Likert-type scale, ranging from 1 “absolutely false” to 7 “absolutely true”. The instrument measures support from family, friends, and significant others, which represent three distinct subscales. The reliability coefficient for each subscale was excellent, ranging from 0.89 and 0.91.

#### 2.3.4. Occupational Stress

Occupational stress was assessed using the Occupational Stress Indicator (OSI; [58,59]). Two scales were taken into account: Sources of Stress and Effects on Health. The former is composed of 61 items distributed into six subscales: Job Factor (JF; 9 items; e.g., “*Having too much work to do*”), Managerial Factor (MF; 11 items; e.g., “*Having personal beliefs in contrast with those of the company*”), Relationships with Others Factor (RF; 10 items; e.g., “*Little encouragement from supervisors*”), Career Factor (CF; 9 items; i.e., “*Holding a position under your ability*”), Home–Work Interface Factor (IF; 11 items; e.g., “*Inability to stop working when you are at home*”), and Organizational Structure Factor (OF; 11 items; e.g., “*Luck of information and involvement in decisions*”). The latter is composed of two subscales, examining the Effects on Health from two perspectives: Psychological (PSY; 18 items; e.g., “*During a working day, do you feel irritated or agitated, though a clear reason does not always seem to be?*”) and Physical (PHY; 12 items; e.g., “*Inability to fall asleep or sleep without interruption*”) Effects. Internal reliability was excellent for each subscale, with Cronbach’s alpha ranging from 0.81 to 0.92.

### 2.4. Statistical Analyses

Descriptive statistical analyses were used to analyze demographic data. Prior to conducting the main analyses, MANOVAs were performed to evaluate whether any significant statistical differences were estimated on the study variables according to gender differences. Mediation analyses were applied to verify whether social support functions as a buffer in the relationship between EI and occupational stress during COVID-19. The process involved examining path a, the association between EI (IV) and social support (M); path b, the impact of social support (M) on occupational stress (DV); and path c and c’, the total and direct effect of EI (IV) on occupational stress (DV). The three sources of social support (family, friends, and significant others) were considered and included in the model as three distinct mediators. Before testing the mediating model, the multivariate normality distribution of data was first examined through the Mahalanobis distance computation. Since the Mardia’s coefficient (192.47) exceeded the critical value associated with twelve-degrees-of-freedom (168), the assumption of multivariate normality was not met. Therefore, we chose to apply the bootstrapping (percentiles) method, a non-parametric resampling procedure recognized as a robust and accurate method for mediation analysis [60] and the best-suited technique to perform when the multivariate normality is violated. IBM SPSS (version 20) and Jamovi (version 1.6.23) were used for the analyses.

## 3. Results

### 3.1. Preliminary Analyses

Prior to conducting the main data analyses, we carried out a correlation inspection between the study variables (see Table 2). Further, MANOVAs were performed to assess the extent to which the scores on the investigated variables differed across genders. The results of MANOVAs showed that no significant statistical differences were estimated between men and women in any of the examined variables (Wilks Λ _(12,352)_ = 0.971, *p* = 0.580, partial η^2^ = 0.029), providing evidence that considering our sample as a whole for further analyses was appropriate. Table 3 depicts the scores obtained by men and women on each variable and the results of the univariate tests.

### 3.2. Mediation Analyses

**Figure 3:**
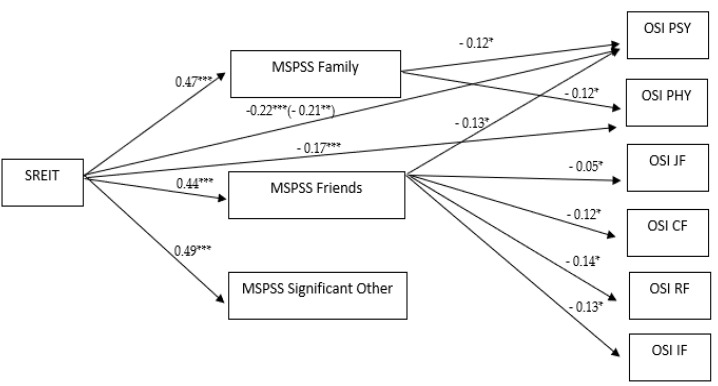
Measurement model testing the relationship between EI and occupational stress through social support. Only significant paths are shown. Direct effects are in parentheses. SREIT: Self-Report Emotional Intelligence Test; MSPSS = Multidimensional Scale of Perceived Social Support; OSI = Occupational Stress Indicator; OSI PSY = Psychological; OSI PHY = Physical; OSI JF = Job Factor; OSI RF = Relational Factor; OSI CF = Career Factor; OSI IF = Work–Home Interface Factor. * *p* < 0.05, ** *p* < 0.01, *** *p* < 0.001.

The significant results for mediation analyses are described in the following section. Table 4 displays all the associations among the investigated variables. Figure 3 depicts the measurement model with only significant paths.

EI showed a significant total (β = −0.22, *p* < 0.001, 95% CI [−0.283, −0.108]) and direct (β = −0.21, *p* = 0.002, 95% CI [−0.305, −0.070]) effect on psychological effects. This relationship also indicated significant effects by adding social support as a mediator. Specifically, support from family and friends functioned as significant mediators, though the magnitude of the association between EI and psychological effects decreased (β = −0.06, *p* = 0.036, 95% CI [−0.097, −0.003], β = −0.05, *p* = 0.027, 95% CI [0.005, 0.092], respectively).

A full mediation was found in the relationship between EI and physical effects (β = −0.17, *p* < 0.001, 95% CI [−0.233, −0.061], as a direct association was not estimated. Only support from family was a significant mediator (β = −0.06, *p* = 0.041, 95% CI [−0.094, −0.002]).

Support from friends mediated the relationship between EI and job factor (β = −0.05, *p* = 0.042, 95% CI [0.001, 0.056]), as well as between EI and relational factor (β = −0.06, *p* = 0.015, 95% CI [0.007, 0.069]), between EI and career factor (β = −0.05, *p* = 0.039, 95% CI [0.001, 0.059]), and between EI and home–work interface factor (β = −0.06, *p* = 0.026, 95% CI [0.004, 0.073]).

Finally, no direct or indirect effects were estimated between EI and managerial factor and between EI and organizational structure factor.

## 4. Discussion

This study’s main objective was to test the direct and indirect relationship between EI and occupational stress, taking into account different sources of social support as mediators. Specifically, we hypothesized that individuals with high EI were more inclined to search for social support and, in turn, tended to experience lower levels of occupational stress. Through the mediation analyses application, all the possible paths were examined, and the associations among the aforementioned variables were verified.

To the best of our knowledge, this is the first attempt addressed to evaluate this mediation model, and the first study in which EI, social support, and occupational stress are jointly examined during the pandemic.

In line with previous research [36,47], our results supported the existence of significant associations between EI and social support, providing evidence that individuals with higher EI tend to perceive greater social support from others. In fact, EI predicted all three sources of social support. This means that individuals able to understand their own and others’ emotions are more likely surrounded by positive and good relationships that strengthen their social competence, and they more easily rely on other people when facing challenging events because they think others are attentive and responsive to their own needs. These findings emphasize how the two concepts are strictly related to each other.

In the current study, three sources of social support were taken into account: social support from family members, friends, and significant others. This can be considered as an innovative aspect of the existing literature on this topic, as the majority of studies on occupational stress mainly focus on the effects of social support from coworkers and supervisors. From this point of view, our findings emphasize that, although the concept of occupational stress is associated with the inability to cope with stress at work, external variables not strictly related to work conditions can also influence the degree of occupational stress. In truth, stress is both a general and complex phenomenon in which multiple variables interact and merge into each other. This suggests that researchers should not limit the investigation of context-dependent stressors, but rather should be aware that other external variables may function both as protective and risk factors. From this perspective, our results are in line with previous research in which not work-related social support positively affected occupational [42,43].

Likewise, occupational stress was assessed considering several aspects of it, i.e., examining the effects on psychological and physical health on the one hand, and on the other, job-related stressors, such as problematic relationships with coworkers and/or supervisors, difficulties in work–home balance, incompatibilities with organizational policies, and issues linked to lacking personal and career development. Specifically, eight facets of occupational stress were identified as outcome variables. Such a distinction allowed us to examine whether the three sources of social support have a diverse impact on the different occupational stress facets and, consequently, whether or not they functioned as a mediator.

In contrast with previous studies [28,29,31] and contrary to our expectations, EI did not report direct effects on occupational stress, except for considering psychological effects as a dependent variable. From this point of view, our results supported the conclusions suggested by some authors [32], according to whom EI is not directly related to stress at the workplace, suggesting that other variables—such as organizational support-—may better predict levels of occupational stress. Another plausible reason for the unexpected direct effects of EI on occupational stress dimensions may be owing to the specific critical period in which data were collected, characterized by the spread of the COVID-19 virus. Despite that EI is intended as a personality trait rather than a temporary state, participants were not adequately instructed to indicate their typical disposition toward understanding managing of their own and others’ emotions. Participants’ responses on some of SREIT items, such as “*I expect good things to happen*” or “*I motivate myself by imagining a good outcome to tasks I take on*”, may be biased owing to the extensive negative emotions experienced during the pandemic.

Nevertheless, significant indirect effects were estimated for almost all occupational stress facets in which social support from family and friends were found to be significant mediators. These findings provide interesting insights for interpretation that may have useful theoretical and practical implications.

From a theoretical perspective, our results indicate the relevance of considering scores on multidimensional measures’ subscales separately. Previous studies on this topic have used the MSPSS for evaluating social support, combining scores obtained in each subscale into a unique total score [37,38,39]. This procedure represents a misuse of multidimensional measures, and we recommend applying it only if a second-order factor analysis has been performed.

In addition, using a total and global score does not allow evaluating whether the different sources of social support have a different impact on occupational stress. Indeed, our analyses’ findings showed that social support from both family and friends has a beneficial effect in minimizing the effects of occupational stress, but social support from significant others did not predict any facets of the outcome variable. These results suggest adopting programs aimed at promoting and reinforcing specific sources of social support, which strengthen social competence and, in turn, have a protective function against maladaptive outcomes.

Any sources of social support had a significant impact on OSI MF and on OSI SF. A viable explanation is that both subscales are strictly related to the specific features of the workplace: the former refers to how individuals perceive others’ expectancies toward themselves, and the latter is the characteristics of the structural and climate organization. Presumably, these aforementioned subscales may be better predicted by a greater sense of social support from coworkers or supervisors, rather than other sources of social support. Future studies may explore this hypothesis.

Although research on occupational stress usually takes into account work-related sources of social support as potential factors affecting or offsetting stress at work, our findings are in line with previous studies outlining how the link between social support and occupational stress is inconsistent and unclear [11]. These inconsistencies may be mainly due to the type of supporters (i.e., source), to the different functions of social support (informational, emotional, and instrumental) considered, and to the specific indicators of occupational stress investigated. From this perspective, additional research is needed that aims at evaluating whether social support (both work- and not work-related social support) differently affect the facets of occupational stress by simultaneously examining the three different functions.

In addition, further studies are also needed to have a deeper understanding of the associations between the selected variables to better justify the proposed model.

As mentioned before, the results of the present study should be considered in light of the critical period in which data were gathered and should be taken with some caution, avoiding generalizations that go beyond the pandemic period. The spread of the COVID-19 virus and the rapid and unexpected changes of habits in daily life and at workplaces may have affected the individual scores on the investigated variables.

### Limitations

Some limitations should be mentioned. First, one concern addresses the missing information about the region in which the participants were living while answering the survey. Indeed, although the current pandemic extended from North to South Italy, during data collection, some regions were in a complete lockdown, whereas others had weaker restrictive measures owing to the virus’s lower incidence rate. This may likely have affected the participants’ response set and their scores on the investigated variables. From this point of view, we are unsure whether our participants can be considered as a representative sample of the Italian population, or whether they better reflect the situation of the country’s specific regions. We suggest future studies address this issue to establish the extent to which the results obtained from the study sample can be generalized to the population to which it refers. Second, we did not explore the influence of other work-related stressors on the degree of occupational stress. For instance, we did not examine how the different changes in working conditions, such as alterations of salary, decrease in working hours, or shifts to teleworking, affected the participants’ perceived level of occupational stress during the pandemic. In fact, a substantial portion of our sample declared that several working conditions changed after the outbreak of the COVID-19 pandemic, but they were taken into account purely at a descriptive level. In particular, we did not investigate the extent to which the transition toward smart working, which implies the need of new organizations and new habits into the family environment, affects occupational stress during the current circumstances. From this perspective, a broader and deeper analysis of any possible stressors generated by the new condition of working at home and by the time and spaces sharing with other family members during a working day should be conducted. Additional research should be aimed at exploring how these stressors influence occupational stress during the global emergency. A further limitation consists of the cross-sectional nature of the study, which prevents us from making inferences on the sequences of events, and there is no information on whether and how the pandemic has changed the associations among the investigated variables. Future longitudinal studies may provide a better knowledge and understanding of the relationships examined.

## 5. Conclusions

The present study represents the first attempt aimed at investigating the mediating role of social support in the relationship between EI and occupational stress, providing a contribution in which the three variables are jointly evaluated during the current global emergency. As the crisis that arose after the spread of the COVID-19 virus is still not under control, neither in Italy nor in other countries in the world, it is imperative to acknowledge which factors influence workers’ stress in the current circumstances. Our findings offer an opportunity to better understand how these variables are related to occupational stress during the pandemic and provide useful insights to design future interventions aimed at ameliorating wellbeing in working contexts. Overall, the results of our study reported that, except for the OSI PSY, which was directly and indirectly predicted by EI, the other facets of occupational stress were negatively associated with EI only through the mediation of social support. Specifically, social support from family and friends showed a protective role in reducing occupational stress. From this perspective, our findings have practical implications, suggesting both health care services and organizations take care of employees’ social relationships and promote and reinforce strong social ties. Specific interventions programs with the purpose of making workers’ social relationships stronger and more solid are highly recommended, with a particular focus on the relationships with family members and friends, as both sources of social support have a relevant function in preventing negative outcomes.

## Figures and Tables

**Table 1:** Descriptive statistics of the study sample.

Demographics	Options	*N*	%
Gender	Males	197	53.7%
	Females	170	46.7%
Marital status	Unmarried	127	34.6%
	Married	177	48.2%
	Divorced	20	5.4%
	Widower	1	0.3%
	Cohabitant	42	11.4%
Parental status	No children	172	47.1%
	1 child	76	20.8%
	2 children	96	26.3%
	3 children	18	4.9%
	4 children	1	0.3%
	5 children	2	0.5%
Educational	Junior high school	42	11.6%
	High school	116	32%
	Degree (Bachelor/Master)	127	35%
	Post-degree	78	21.5%
Employment status	Private sector	240	65.8%
	Public sector	100	27.4%
	Tertiary sector	25	6.8%
Way of working during COVID-19	work remotely only	80	21.8%
	work remotely, but also at the workplace	99	27%
	work at the workplace, but also remotely,	63	17.2%
	work at the workplace only	120	32.7%
Amount of changes in working conditions during COVID-19	Not at all	51	13.9%
	A little	135	13.9%
	Somewhat	101	27.5%
	Very much	64	17.4%
Working conditions changed during COVID-19	Salary increase	29	5%
	Salary decrease	78	13.4%
	Working hours increase	126	21.6%
	Working hours decrease	69	11.8%
	Kind of job activity	69	11.8%
	Layoff	26	4.5%
	Relationships with coworkers	118	20.2%
	Relationships with supervisors	49	8.4%
	Other	19	3.3%

**Table 2:** Correlations between the study variables.

Variable	1	2	3	4	5	6	7	8	9	10	11	12
1. SREIT	-											
2. MSPSS Family	0.47 **	-										
3. MSPSS Friends	0.44 **	0.48 **	-									
4. MSPSS Significant Other	0.49 **	0.51 **	0.52 **	-								
5. OSI PSY	−0.22 **	−0.17 **	−0.12 *	0.03	-							
6. OSI PHY	−0.17 **	−0.19 **	−0.08	0.09	0.65 **	-						
7. OSI JF	−0.01	−0.04	0.07	0.01	0.55 **	0.55 **	-					
8. OSI MF	0.10	0.07	−0.08	−0.09	0.38 **	0.40 **	0.81 **	-				
9. OSI RF	0.01	0.02	−0.10 *	−0.01	0.47 **	0.50 **	0.83 **	0.84 **	-			
10. OS CF	0.03	0.01	−0.10 *	0.04	0.44 **	0.46 **	0.80 **	0.83 **	0.82 **	-		
11. OSI IF	0.09	0.10	−0.15 *	0.07	0.41 **	0.40 **	0.80 **	0.85 **	0.80 **	0.80 **	-	
12. OSI SF	0.10	0.07	0.09	0.09	0.32 **	0.37 **	0.77 **	0.89 **	0.81 **	0.81 **	0.83 **	-

Note: SREIT: Self-Report Emotional Intelligence Test; MSPSS = Multidimensional Scale of Perceived Social Support; OSI = Occupational Stress Indicator; OSI_PSY = Psychological; OSI PHY = Physical; OSI JF = Job Factor; OSI MF = Managerial Factor; OSI RF = Relational Factor; OSI CF = Career Factor; OSI IF = Work–Home Interface Factor; OSI SF = Organizational Structure Factor. * *p* < 0.05; ** *p* < 0.01.

**Table 3:** Scores obtained by men and women on the study variables.

	Gender	*M*	*SD*	*F*	*Sig*	*Partial η* ^2^
STREIT	M	116.63	16.32			
	F	116.41	15.85			
	Tot	116.53	16.08	0.018	0.895	0.000
MPSS Family	M	22.86	5.73			
	F	23.49	5.01			
	Tot	23.15	5.41	1.21	0.272	0.003
MPSS Friends	M	21.49	5.71			
	F	21.57	5.85			
	Tot	21.53	5.77	0.017	0.896	0.000
MPSS Significant Other	M	23.29	5.24			
	F	23.33	5.63			
	Tot	23.31	5.42	0.005	0.946	0.000
OSI PSY	M	55.52	15.32			
	F	56.33	12.41			
	Tot	55.89	14.04	0.301	0.584	0.001
OSY PHY	M	32.80	14.79			
	F	33.63	12.45			
	Tot	33.18	13.75	0.333	0.564	0.001
OSI JF	M	30.45	9.63			
	F	30.60	7.97			
	Tot	30.52	8.89	0.026	0.873	0.000
OSI MF	M	39.32	11.82			
	F	40.34	11.23			
	Tot	39.79	11.55	0.706	0.401	0.002
OSI RF	M	34.28	10.38			
	F	33.76	9.15			
	Tot	34.04	9.82	0.251	0.617	0.001
OSI CF	M	32.50	9.56			
	F	32.43	8.81			
	Tot	32.47	9.21	0.006	0.939	0.000
OSI IF	M	39.86	11.03			
	F	40.30	11.02			
	Tot	40.06	11.01	0.141	0.708	0.000
OSI SF	M	40.56	11.70			
	F	40.73	11.81			
	Tot	40.64	11.74	0.020	0.888	0.000

Note: M = males; F = females; SREIT: Self-Report Emotional Intelligence Test; MSPSS = Multidimensional Scale of Perceived Social Support; OSI = Occupational Stress Indicator; OSI PSY = Psychological; OSI PHY = Physical; OSI JF = Job Factor; OSI MF = Managerial Factor; OSI RF = Relational Factor; OSI CF = Career Factor; OSI IF = Work–Home Interface Factor; OSI SF = Organizational Structure Factor.

**Table 4:** Relationships between emotional intelligence (EI), social support, and occupational stress.

		95% CI			
Type	Effect	*LL*	*UP*	β	*p*
Indirect	SREIT → MSPSS Family → OSI PSY SREIT → MSPSS Friends → OSI PSY SREIT → MSPSS Significant Other → OSI PSY	−0.097 0.005 −0.055	−0.003 0.091 0.042	−0.06 −0.05 0.00	0.036 0.027 0.800
Direct	SREIT → OSI PSY	−0.305	−0.070	−0.21	0.002
Total	SREIT → OSI PSY	−0.283	−0.108	−0.22	<0.001
Indirect	SREIT → MSPSS Family → OSI PHY SREIT → MSPSS Friends → OSI PHY SREIT → MSPSS Significant Other → OSI PHY	−0.094 −0.026 −0.083	−0.002 0.056 0.014	−0.06 0.01 −0.04	0.041 0.474 0.163
Direct	SREIT → OSI PHY	−0.195	0.036	−0.09	0.177
Total	SREIT → OSI PHY	−0.233	−0.061	−0.17	<0.001
Indirect	SREIT → MSPSS Family → OSI MF SREIT → MSPSS Friends → OSI MF SREIT → MSPSS Significant Other → OSI MF	−0.047 −0.011 −0.014	0.029 0.059 0.068	−0.01 0.03 0.04	0.643 0.175 0.192
Direct	SREIT → OSI MF	−0.072	0.123	0.04	0.609
Total	SREIT → OSI MF	−0.004	0.141	0.09	0.066
Indirect	SREIT → MSPSS Family → OSI JB SREIT → MSPSS Friends → OSI JF SREIT → MSPSS Significant Other → OSI JF	−0.052 0.001 −0.034	0.007 0.056 0.029	−0.04 −0.05 0.01	0.139 0.042 0.867
Direct	SREIT → OSI JF	−0.079	0.071	.01	0.913
Total	SREIT → OSI JF	−0.057	0.056	.01	0.980
Indirect	SREIT → MSPSS Family → OSI RF SREIT → MSPSS Friends → OSI RF SREIT → MSPSS Significant Other → OSI RF	−0.035 0.007 −0.055	0.029 0.069 0.015	−0.01 −0.06 −0.03	0.862 0.015 0.254
Direct	SREIT → OSI RF	−0.095	0.072	−0.02	0.784
Total	SREIT → OSI RF	−0.059	0.066	0.01	0.915
Indirect	SREIT → MSPSS Family → OSI CF SREIT → MSPSS Friends → OSI CF SREIT → MSPSS Significant Other → OSI CF	−0.046 0.001 −0.034	0.016 0.059 0.032	−0.03 −0.06 −0.01	0.344 0.039 0.942
Direct	SREIT → OSI CF	−0.075	0.082	0.01	0.922
Total	SREIT → OSI CF	−0.040	0.077	0.03	0.543
Indirect	SREIT → MSPSS Family → OSI IF SREIT → MSPSS Friends → OSI IF SREIT → MSPSS Significant Other → OSI IF	−0.024 0.004 −0.049	0.049 0.073 0.029	0.02 −0.05 −0.01	0.491 0.026 0.607
Direct	SREIT → OSI IF	−0.074	0.112	0.03	0.700
Total	SREIT → OSI IF	−0.009	0.129	0.09	0.092
Indirect	SREIT → MSPSS Family → OSI SF SREIT → MSPSS Friends → OSI SF SREIT → MSPSS Significant Other → OSI SF	−0.043 −0.062 −0.034	0.035 0.056 0.049	−0.01 0.02 0.01	0.837 0.278 0.738
Direct	SREIT → OSI SF	−0.029	0.170	0.09	0.167
Total	SREIT → OSI SF	−0.019	0.167	0.01	0.233

Note: SREIT: Self-Report Emotional Intelligence Test; MSPSS = Multidimensional Scale of Perceived Social Support; OSI = Occupational Stress Indicator; OSI PSY = Psychological; OSI PHY = Physical; OSI JF = Job Factor; OSI MF = Managerial Factor; OSI RF = Relational Factor; OSI CF = Career Factor; OSI IF = Work–Home Interface Factor; OSI SF = Organizational Structure Factor. CI = confidence intervals; LL = lower limit; UL = upper limit.

## Data Availability

The data presented in this study are available on request from the corresponding author.
